# Signaling mechanism for modulation by ATP of glycine receptors on rat retinal ganglion cells

**DOI:** 10.1038/srep28938

**Published:** 2016-06-30

**Authors:** Ping-Ping Zhang, Gong Zhang, Wei Zhou, Shi-Jun Weng, Xiong-Li Yang, Yong-Mei Zhong

**Affiliations:** 1 Institutes of Brain Science, State Key Laboratory of Medical Neurobiology and Collaborative Innovation Center for Brain Science, Fudan University, 138 Yixueyuan Road, Shanghai 200032, China

## Abstract

ATP modulates voltage- and ligand-gated channels in the CNS via the activation of ionotropic P2X and metabotropic P2Y receptors. While P2Y receptors are expressed in retinal neurons, the function of these receptors in the retina is largely unknown. Using whole-cell patch-clamp techniques in rat retinal slice preparations, we demonstrated that ATP suppressed glycine receptor-mediated currents of OFF type ganglion cells (OFF-GCs) dose-dependently, and the effect was in part mediated by P2Y_1_ and P2Y_11_, but not by P2X. The ATP effect was abolished by intracellular dialysis of a G_q/11_ protein inhibitor and phosphatidylinositol (PI)-phospholipase C (PLC) inhibitor, but not phosphatidylcholine (PC)-PLC inhibitor. The ATP effect was accompanied by an increase in [Ca^2+^]_i_ through the IP_3_-sensitive pathway and was blocked by intracellular Ca^2+^-free solution. Furthermore, the ATP effect was eliminated in the presence of PKC inhibitors. Neither PKA nor PKG system was involved. These results suggest that the ATP-induced suppression may be mediated by a distinct G_q/11_/PI-PLC/IP_3_/Ca^2+^/PKC signaling pathway, following the activation of P2Y_1,11_ and other P2Y subtypes. Consistently, ATP suppressed glycine receptor-mediated light-evoked inhibitory postsynaptic currents of OFF-GCs. These results suggest that ATP may modify the ON-to-OFF crossover inhibition, thus changing action potential patterns of OFF-GCs.

As a neurotransmitter in the CNS, ATP functions by acting on two distinct subfamilies of P2 purinoceptors: seven ionotropic P2X receptors (P2X_1-7_) and eight metabotropic mammalian P2Y receptors (P2Y_1,2,4,6,11,12,13,14_)^1^,^2^. These receptors are involved in regulating voltage-gated Ca^2+^, K^+^ channels, ligand-gated NMDA channels^3^,^4^,^5^,^6^,^7^,^8^,^9^,^10^ and neurotransmitter release^11^,^12^,^13^. Moreover, ATP may be hydrolyzed to adenosine by ecto-ATPases and ectonucleotidases^14^, which regulates neuronal activity by activating neuronal adenosine receptors (P1 purinoceptors)^15^,^16^.

Expression of P2 receptors has been described in rat retinal neurons and Müller cells^17^,^18^,^19^,^20^,^21^,^22^,^23^,^24^,^25^. In the retina, ATP released by Müller cells may act on both neurons and Müller cells^15^,^16^. In the inner retina, another source of ATP is cholinergic amacrine cells (ACs)^26^,^27^. In addition, the enzymes required for deactivating extracellular ATP are also found in the synaptic layers of the rat retina^19^. It is therefore highly possible that ATP may modulate the activity of retinal neurons.

Ganglion cells (GCs) are output neurons in the retina. Functionally, GCs are classified into ON and OFF subtypes according to distinct features of their light responses^28^,^29^. While ON and OFF pathways process visual signals in a relatively independent manner, their signals may interact with each other at multiple levels^30^,^31^,^32^,^33^,^34^,^35^,^36^,^37^,^38^,^39^. For instance, in the inner retina cumulative evidence suggests that the so-called ON-to-OFF pathway crossover inhibition, mediated by glycinergic ACs, including AII ACs, plays a crucial role in the interplay between ON and OFF pathways^30^,^31^,^32^,^33^,^34^,^35^,^36^,^37^,^38^,^39^. It is known that AII ACs modulate the firing rates of OFF type GCs (OFF-GCs) by sending direct glycinergic signal to these cells^32^,^35^,^36^,^40^. ATP has been found to modulate the activity of GCs. ATP released from Müller cells evokes hyperpolarizing responses and outward currents in a subset of GCs, thus providing an inhibition of the firing rate of these cells^15^,^16^. Of interest, ATP-induced modulatory actions on the activity of GCs differ between the ON and OFF pathways^41^.

Because of the importance of OFF-GCs in the ON-to-OFF crossover inhibition and the essential role of glycinergic signal in shaping temporal features of OFF-GC responses, the present work focused on ATP-induced modulation of glycine-receptor mediated responses of OFF-GCs. By using whole-cell patch-clamp techniques in rat retinal slice preparations, we characterized how ATP modulated glycine currents of OFF-GCs, by activating P2Y receptors and explored the intracellular signaling pathway mediating such a modulation. Our results clearly show that a distinct G_q/11_/phosphatidylinostiol (PI)-phospholipase C (PLC)/inositol-1,4,5-trisphosphate (IP_3_)/Ca^2+^/protein kinase C (PKC) signaling pathway is responsible for the ATP effect. Consistent with this, we also found that ATP suppressed light-evoked glycine receptor-mediated inhibitory postsynaptic currents (L-IPSCs) of OFF-GCs via P2Y receptors.

## Results

### ATP suppresses glycine currents of OFF-GCs

**Figure 1:**
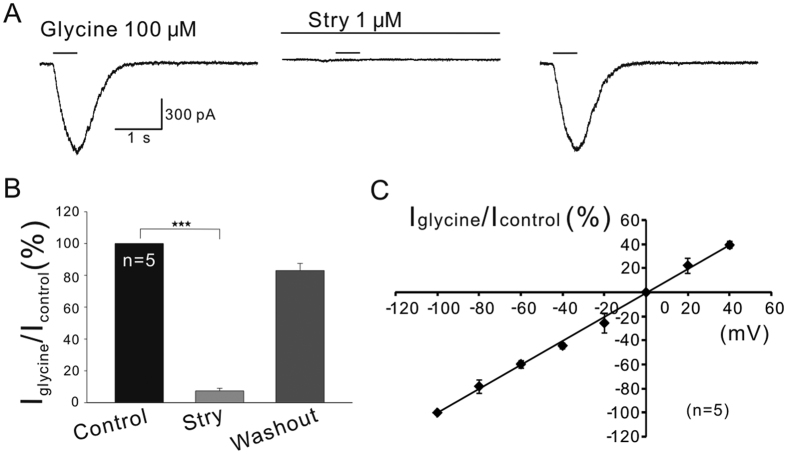
Characterization of glycine receptor-mediated currents recorded in rat GCs. (**A**) Representative current response of a GC clamped at −60 mV, which was elicited by local puff (500 ms) of 100 μM glycine at intervals of 2 min to the dendrites in a rat retinal slice. The current was reversibly suppressed by 1 μM strychnine (Stry). The time- and amplitude-scale bars shown below the current trace are for the current responses. Durations of drug applications are indicated by horizontal lines above the current traces. (**B**) Bar chart summarizing the effects of 1 μM strychnine on glycine currents of GCs (n = 5). ***P < 0.001 *vs.* control. (**C**) Average current-voltage relationship of glycine-induced currents from 5 GCs. Current responses for each cell at different holding potentials were normalized to the response obtained at −100 mV. The data are presented as means ± SEM in all figures.

We first characterized glycine-induced currents in rat GCs. Glycine receptor-mediated currents were pharmacologically isolated by adding D-AP5, CNQX, bicuculline and TTX to bath Ringer’s (see Methods for details). Figure 1A shows that the current of a GC clamped at −60 mV, which was induced by local puff of 100 μM glycine to the dendrites of the cell in Ringer’s containing the above antagonists. The current was almost completely abolished by 1 μM strychnine, a specific antagonist of glycine receptors^42^ (7.36 ± 1.62% of control, n = 5, *P* < 0.001; Fig. 1B). The current response returned to the control level after washout with Ringer’s (83.0 ± 4.42% of control, *P* > 0.05). The current-voltage relationship of the currents was linear, with a reversal potential of 0.15 ± 1.4 mV (n = 5; Fig. 1C), which is very close to E_Cl_- (0 mV), calculated according to the Nernst equation.

**Figure 2:**
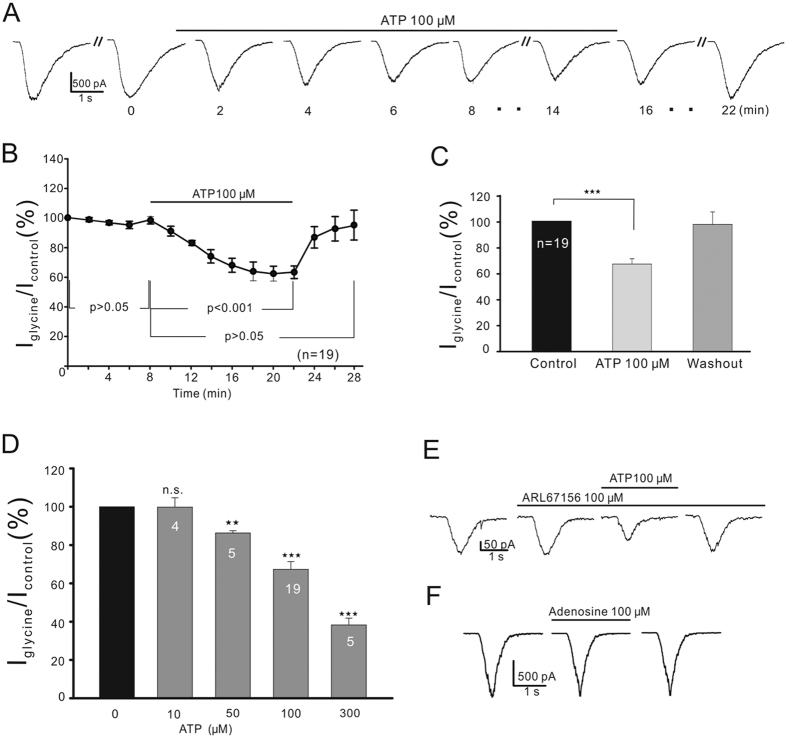
ATP suppresses glycine currents of OFF-GCs. (**A**) Representative recordings, showing the effect of 100 μM ATP on glycine currents of an OFF-GC. Drug application is indicated by the horizontal line above the current traces and the times, at which the current traces were recorded, are marked below (min). (**B**) Average peak amplitudes of glycine currents are plotted as a function of time, showing that ATP suppressed the glycine currents. (**C**) Bar chart showing the effect of 100 μM ATP on glycine current amplitudes of OFF-GCs (n = 19). (**D**) ATP suppressed the glycine currents in a dose-dependent manner. Cell numbers are indicated inside the bars. (**E**) ATP persisted to suppress the glycine current in the presence of 100 μM ARL67156. (**F**) Adenosine of 100 μM did not change the glycine current. **P < 0.01, ***P < 0.001 *vs.* control. n.s., represents no significant difference.

Application of 100 μM ATP elicited no detectable current in OFF-GCs (data not shown). When 100 μM ATP was bath-applied, as shown in Fig. 2A, the current induced by 100 μM glycine was suppressed in a progressive manner during the first 6 min after ATP application, and the current became stable in about 8 min and was kept at a similar level thereafter. ATP-induced suppression of glycine currents was observed in most of the OFF-GCs tested (19 out of 23, 82.61%). The average current amplitudes, following 14 min perfusion of 100 μM ATP, were reduced to 67.3 ± 4.05% of control (n = 19, *P* < 0.001; Fig. 2B,C). In the remaining four cells, ATP had no effects on the glycine currents (4/23, 17.39%).

We further examined the concentration dependence of the ATP effect. For these experiments, data were pooled only from the cells, in which peak amplitudes to 100 μM glycine applied at intervals of 2 min were altered less than 5% during a period of 8 min prior to the experiment. For each cell, only a single concentration of ATP was tested, and the response amplitude obtained after 14-min incubation of ATP was normalized to the level recorded prior to the incubation (control). Following the 8-min washout, the response commonly returned to the control level (Fig. 2C). The data were discarded in case the response amplitudes (after 8-min washout) were changed by more than 5% of control. Figure 2D shows how the ATP effect depended upon ATP concentration. No suppression was seen with 10 μM ATP (104.2 ± 4.67%, n = 4, *P* > 0.05), but the glycine currents were respectively reduced to 86.3 ± 1.18% (n = 5, *P* < 0.01), 67.3 ± 4.05% (n = 19, *P* < 0.001) and 38.3 ± 3.53% (n = 5, *P* < 0.001) of control following ATP incubation at concentrations of 50 μM, 100 μM and 300 μM. Based on these data, ATP of 100 μM was chosen for all experiments to be subsequently described.

Since extracellular ATP can be converted into adenosine by ectonucleotidases^14^, we tested whether ATP or adenosine induced the suppression. ARL67156 (100 μM), an ectonucleotidase antagonist, which blocks the degradation of ATP^43^, did not change the glycine currents of OFF-GCs (105.1 ± 2.33% of control, n = 7, *P* > 0.05). In the presence of ARL67156, application of ATP still suppressed the glycine currents to an extent (67.5 ± 5.0% of control, n = 7, *P* < 0.001; Fig. 2E), comparable to that obtained in Ringer’s. Moreover, perfusion of adenosine (100 μM) had no effect on the glycine currents of OFF-GCs (98.4 ± 0.48% of control, n = 6, *P* > 0.05; Fig. 2F). These results indicate that it was ATP, but not the hydrolysed products of ATP, suppressed glycine currents of OFF-GCs.

### ATP effect is mediated by P2-purinoceptors

**Figure 3:**
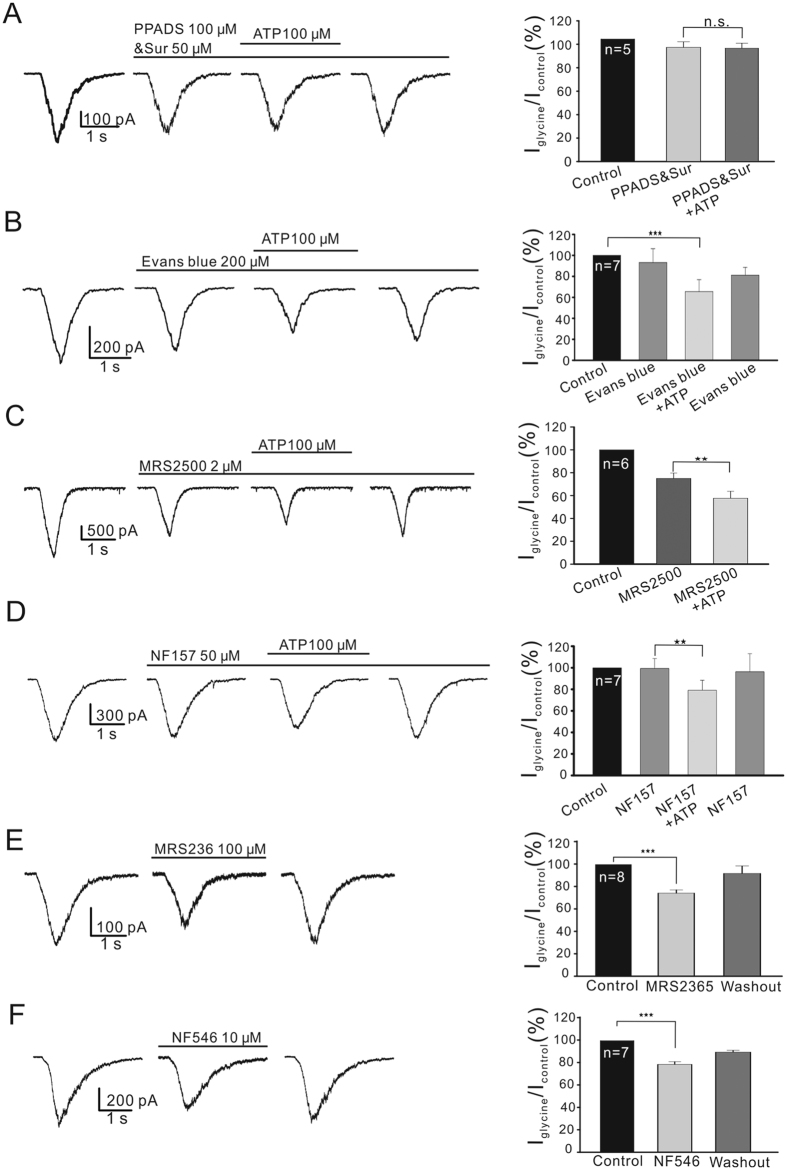
ATP-induced suppression of glycine currents in OFF-GCs is mediated by P2 receptors. (**A**) Representative current traces, taken from an OFF-GC, showing that during perfusion of PPADS (100 μM) and suramin (Sur) (50 μM), application of ATP (100 μM) no longer suppressed the glycine current. (**B**) Current traces of an OFF-GC, showing that in the presence of Evans blue (200 μM), 100 μM ATP still suppressed the glycine current. (**C**,**D**) Representative recordings obtained from two different OFF-GCs, showing that in the presence of 2 μM MRS2500 (**C**) or 50 μM NF157 (**D**), ATP persisted to suppress the glycine currents. Note that extracellular application of 2 μM MRS2500 per se suppressed the current (**C**), and the ATP-induced suppression of glycine currents was partially attenuated by MRS2500/NF157. **(E**,**F**) Current traces of two different OFF-GCs, showing that application of 100 μM MRS2365 (**E**) or 10 μM NF546 (**F**) suppressed the glycine currents. Each bar chart in A-F shows the statistical analysis of the data. ***P < 0.001 *vs.* control (**B**,**E**,**F**); **P < 0.01, as compared to the current amplitudes before ATP application (**C**,**D**).

Figure 3A shows that, in the presence of PPADS (a relatively selective antagonist of homomeric P2X_1,7_, P2Y_1,2,4_) and suramin (a relative selective antagonist of P2X_1,2,3,5,7_, P2Y_2,4,11_)^41^,^44^,^45^, ATP application for 8 min failed to affect the glycine currents with an average being 94.2 ± 4.27% of control (*P* > 0.05). To explore which P2-receptor subtype(s) (P2X, P2Y) was (were) involved, we first perfused Evans blue (200 μM), a broad spectrum antagonist of P2X receptors^44^,^46^, which exerted no effect on glycine currents (93.1 ± 3.22% of control, *P* > 0.05), but co-application of ATP suppressed the currents to 65.5 ± 1.36% of control (n = 7, *P* < 0.001; Fig. 3B). This result is suggestive of no involvement of P2X receptors in the ATP effect.

P2Y_1,2,4,6_^18^,^21^,^22^,^23^,^24^,^25^ and P2Y_11_ (Zhang and Zhong, unpublished data) are expressed in rat GCs. There are two P2Y antagonists now commercially available, MRS2500, a selective P2Y_1_ antagonist^47^ and NF157, a selective P2Y_11_ antagonist^48^. Perfusion of 2 μM MRS2500 reduced the glycine currents, and the currents became stable in about 8 min (74.9 ± 4.78% of control, n = 6; Fig. 3C). During the perfusion of MRS2500, addition of ATP further suppressed the currents (Fig. 3C) and the current amplitudes obtained after an 8-min perfusion of ATP were 76.9 ± 2.35% of those obtained before ATP perfusion (*P* < 0.01 *vs.* ATP), which were less than the results obtained in normal Ringer’s (67.3 ± 4.05%). No significant change in suppression was seen with a higher concentration (20 μM) of MRS2500 (data not shown). NF157 (50 μM), while applied alone, produced no change in glycine currents (99.3 ± 9.12%, n = 7, *P* > 0.05), and co-application of ATP suppressed the currents to 79.1 ± 9.28% of those obtained before ATP perfusion (*P* < 0.05 *vs.* ATP; Fig. 3D), which were also less than the results obtained in normal Ringer’s (67.3 ± 4.05%). It is likely that blockade of P2Y_1_ or P2Y_11_ partially attenuated the ATP-induced suppression of glycine currents. Consistently, application of 100 μM MRS2365, an agonist of P2Y_1_^49^, and 10 μM NF546, an agonist of P2Y_11_^50^, respectively suppressed the glycine currents of OFF-GCs to 74.2 ± 2.91% (for MRS2365) (n = 8, P < 0.001; Fig. 3E) or to 78.5 ± 2.35% (for NF546) (n = 7, P < 0.001; Fig. 3F), thus mimicking the ATP effect. All these results imply that both P2Y_1_ and P2Y_11_ partially mediated the ATP effect.

**Figure 4:**
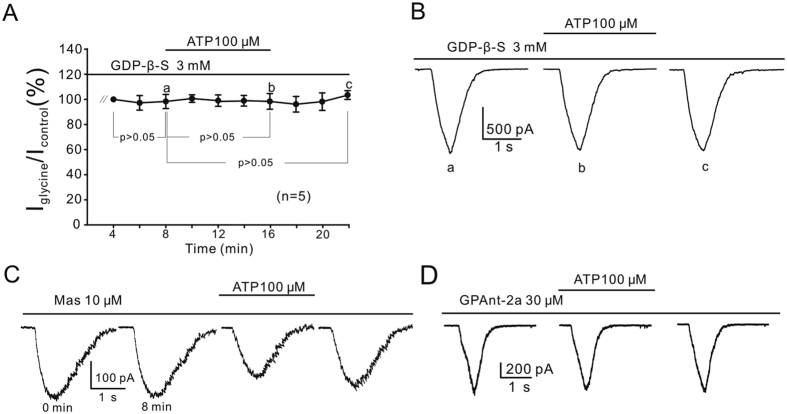
G-protein mediates the suppression of glycine currents by ATP. (**A**) Changes in glycine currents of OFF-GCs caused by ATP are plotted as a function of time during internal infusion of 3 mM GDP-β-S. The data obtained for each cell were normalized to the amplitudes obtained at 8 min after GDP-β-S infusion when the currents became stable. (**B**) Representative current responses recorded at times indicated by a, b and c shown in (**A**). (**C**) Representative recordings of an OFF-GC, showing that during the internal infusion of 10 μM mastoparan (Mas), application of ATP still suppressed the glycine current. (**D**) Current traces of an OFF-GC, showing that in the presence of 30 μM GPAnt-2a, ATP failed to suppress the glycine current.

Given P2Y receptors being G-protein-coupled^44^,^45^, the ATP effect should be eliminated when G-protein activity is inhibited. This was experimentally demonstrated. ATP did not suppress glycine currents recorded from OFF-GCs which were intracellularly dialyzed with the G-protein inhibitor GDP-β-S (3 mM) for more than 8 min (98.4 ± 6.25% of control, n = 5, *P* > 0.05; Fig. 4A,B). Recent evidence suggests that P2Y_1,2,4,6,11_ and P2Y_12-14_ are mainly coupled to G_q/11_ and G_i/o_ proteins, respectively^51^,^52^. We further examined which subtype(s) of G-proteins may mediate the ATP effect. Internal dialysis with 10 μM mastoparan, a peptide activator of G_i_ and G_o_^53^, for 8 min appeared to slow down the decay phases of the glycine currents of OFF-GCs, but did not change glycine current amplitudes (100.7 ± 3.69% of control, n = 5, *P* > 0.05), and addition of ATP persisted to suppress the currents to 63.7 ± 7.66% of control (*P* < 0.001; Fig. 4C). In contrast, during internal infusion of 30 μM GPAnt-2a, a specific G_q/11_ protein inhibitor^51^, application of ATP no longer suppressed the glycine currents of OFF-GCs (95.3 ± 2.48% of control, n = 9, *P* > 0.05; Fig. 4D).

### PI-PLC, but not PC-PLC, signaling pathway mediates ATP-induced suppression of glycine currents

**Figure 5:**
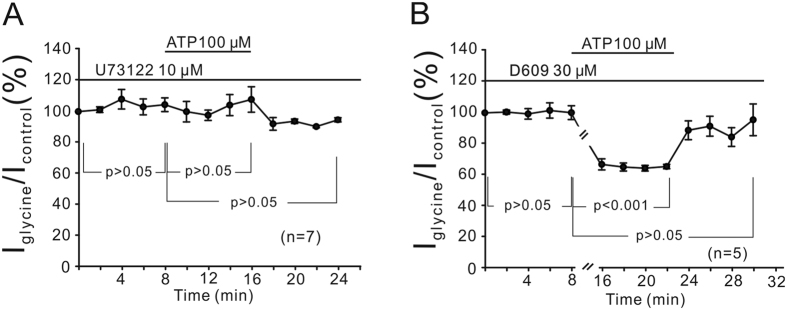
PI-PLC, but not PC-PLC, is involved in modulation by ATP of glycine currents. (**A**) Average peak amplitudes of glycine currents are plotted as a function of time, showing that the effective inhibition of endogenous PI-PLC by U73122 eliminated ATP-induced suppression of glycine currents. (**B**) Plot of average peak glycine current amplitudes as a function of time, showing that ATP-induced suppression of glycine currents was still seen in the presence of 30 μM D609.

Activation of P2Y receptors could regulate several second messengers, and the PLC-PKC signaling pathway is a major downstream effector following P2Y receptor activation^52^,^54^,^55^,^56^. To test whether the PLC pathway may be involved, we investigated how U73122 (PI-PLC inhibitor) or D609 (PC-PLC inhibitor)^57^ changed the ATP effect. Figure 5A shows that internal infusion of 10 μM U73122 for 8 min did not change glycine currents of OFF-GCs (104.4 ± 4.40%, n = 7, *P* > 0.05), then addition of ATP for 8 min no longer reduced the currents (107.6 ± 8.18%, *P* > 0.05). In contrast, during internal infusion of 30 μM D609, application of ATP reduced the currents to 65.5 ± 1.42% (n = 5, *P* < 0.001; Fig. 5B) of those obtained before the ATP application. These results suggest the involvement of the PI-PLC pathway, but not the PC-PLC one.

### Intracellular Ca^2+^ is involved in ATP-induced suppression of glycine currents

**Figure 6:**
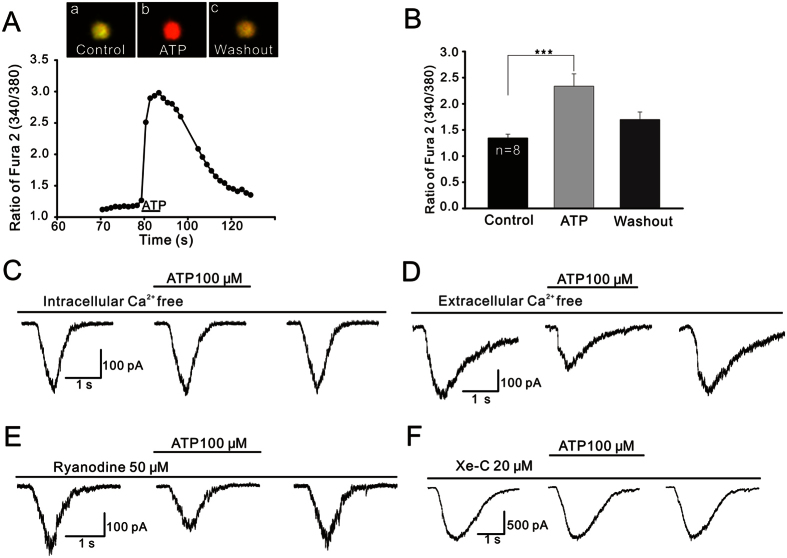
Calcium relevance of ATP-induced suppression of glycine currents. (**A**) A continuous recording of [Ca^2+^]_i_ in a GC, represented by the ratio of fura-2 AM fluorescence at 340 nm and 380 nm (340/380). Application of ATP dramatically increased [Ca^2+^]_i_ in a reversible manner. Three CCD images of an another GC loaded with fura-2 AM were taken before (a) and 10 s following ATP perfusion (b), and after washout (c). (**B**) Bar chart showing the ATP-caused changes in [Ca^2+^]_i_ in GCs. ***P < 0.001 *vs.* control. (**C**) Representative recordings from an OFF-GC, showing that during internal infusion of Ca^2+^-free solution (containing 10 mM BAPTA), ATP failed to suppress the glycine current. (**D**) Representative recordings from an OFF-GC, showing that ATP still suppressed the glycine current in Ca^2+^-free extracellular solution (containing 1 mM EGTA). (**E**,**F**) Current traces of two OFF-GCs, showing that during the internal infusion of ryanodine (50 μM) (**E**), but not Xe-C (20 μM) (**F**), the ATP suppression effect on glycine current was seen.

As Ca^2+^ is considered to be a mediator between PI-PLC and PKC^58^, whether the ATP effect is dependent on [Ca^2+^]_i_ was further examined. We first monitored ATP-induced changes in [Ca^2+^]_i_ in isolated GCs via calcium imaging. Because it is impossible to make a distinction between ON- and OFF-GC when they were isolated, the cells on which calcium imaging was performed should contain both the types. As shown in a representative result (Fig. 6A), application of ATP induced a significant increase in [Ca^2+^]_i_ of the GC in a reversible manner, represented as the ratio of fura-2 (340/380). The CCD images (Fig. 6a–c) show the changes in [Ca^2+^]_i_ induced by ATP in the soma of a GC. In eight GCs tested the averaged peak ratio value of fura-2 (340/380) obtained with the perfusion of ATP was 2.34 ± 0.23, which was significantly higher compared with the value obtained in Ringer’s (1.25 ± 0.07, *P* < 0.001; Fig. 6B). Consistently, internal infusion of Ca^2+^-free solution containing 10 mM BAPTA, a calcium chelator^59^, the application of ATP for 8 min no longer suppressed the glycine currents (95.9 ± 2.49% of control, n = 5, *P* > 0.05; Fig. 6C). ATP-induced increase in [Ca^2+^]_i_ may be induced by an increase in extracellular Ca^2+^ influx across the plasma membrane via Ca^2+^ channels and/or an increase in Ca^2+^ release from intracellular calcium stores. When OFF-GCs were bathed in Ca^2+^-free extracellular solution containing 1 mM EGTA, a calcium chelator^60^, ATP still suppressed the glycine currents to 62.1 ± 1.56% of control (n = 6, *P* < 0.001; Fig. 6D), suggesting that the ATP effect was independent of changes in extracellular calcium levels ([Ca^2+^]_o_).

Ca^2+^ release from intracellular calcium stores could be mediated by ryanodine- and/or IP_3_-sensitive pathways. With intracellular dialysis of 50 μM ryanodine, which depletes ryanodine-sensitive calcium sites^61^, ATP still significantly suppressed the currents (64.1 ± 3.64% of control, n = 6, *P* < 0.001; Fig. 6E). In contrast, during internal infusion of 20 μM xestospongin-C (Xe-C), an IP_3_ receptor antagonist, addition of ATP no longer suppressed the glycine currents of OFF-GCs (97.9 ± 5.81% of control, n = 5, *P* > 0.05; Fig. 6F). Similar results were obtained with internal infusion of heparin (5 mg/ml), another IP_3_ receptor antagonist (data not shown).

### Role of PKC activity

**Figure 7:**
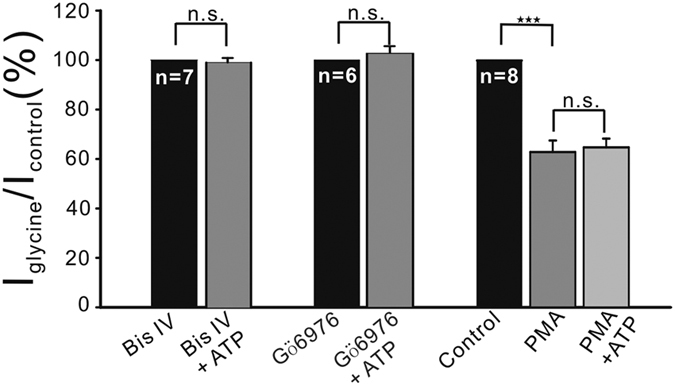
ATP-induced suppression of glycine currents in OFF-GCs is PKC-dependent. Bar charts, showing that ATP failed to suppress the glycine currents during internal infusion of 10 μM Bis-IV or 30 μM Gö6976. Extracellular application of 1 μM PMA decreased the glycine currents and in the presence of PMA ATP application did not further suppress the currents.

Changes in [Ca^2+^]_i_ are known to modulate the activity of PKC^62^. The effects of two PKC inhibitors on ATP-induced suppression of glycine currents in OFF-GCs were examined. None of the seven cells, tested with pipette solution containing 10 μM bisindolylmaleimide IV (Bis-IV), a general inhibitor of PKC, responded to application of ATP (99.3 ± 1.83% of control, *P* > 0.05; Fig. 7). Internal infusion of 2 μM Gö6976, an inhibitor of conventional Ca^2+^-dependent PKCα and β1 isozymes, yielded a similar result. That is, in the presence of Gö6976 the mean current amplitude during ATP perfusion was hardly changed (102.7 ± 2.61% of control, n = 6, *P* > 0.05; Fig. 7). Moreover, perfusion of 1 μM PMA, a PKC activator, suppressed the glycine currents in a progressive manner, with the amplitudes at 8 min being 62.8 ± 4.68% of control (n = 8, *P* < 0.001; Fig. 7), thus mimicking the ATP effect. During the perfusion of PMA, ATP did not cause a further suppression of the currents (103.9 ± 2.59% of the currents obtained before ATP application, *P* > 0.05 *vs.* ATP).

### No involvement of cAMP-PKA and cGMP-PKG signaling pathways in the action of ATP

Finally, we tested whether cAMP-PKA and cGMP-PKG signaling pathways may be involved in the ATP effect on OFF-GCs. During intracellular application of 3 mM cAMP or 4 mM cGMP, ATP still suppressed the glycine currents in all OFF-GCs tested (65.7 ± 2.38% of control, n = 5, *P* < 0.001 for cAMP; 64.3 ± 9.21% of control, n = 5, *P* < 0.001 for cGMP). Furthermore, internal infusion of Rp-cAMP (50 μM), a PKA inhibitor or KT5823 (30 μM), a PKG inhibitor, did not change the ATP effect on glycine currents respectively (62.1 ± 5.09% for Rp-cAMP, n = 6, *P* < 0.001; 65.1 ± 3.42% for KT5823, n = 5, *P* < 0.001). These results suggest the involvement of neither cAMP-PKA nor cGMP-PKG pathway.

### ATP suppresses glycine receptor-mediated L-IPSCs of OFF-GCs through P2Y receptors

**Figure 8:**
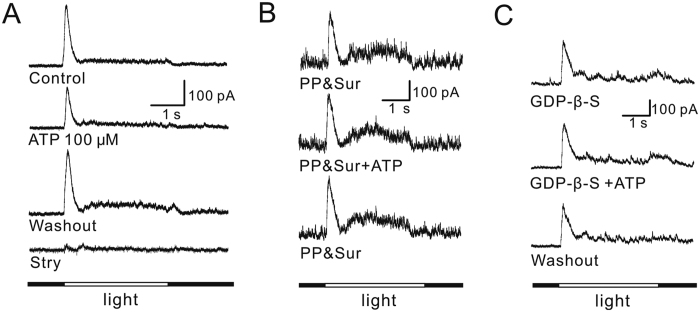
ATP suppresses glycine receptor-mediated L-IPSCs of OFF-GCs via P2Y receptor activation. (**A**) L-IPSCs from an OFF-GC, clamped at 0 mV, were elicited by a 3-second, full-field light stimulus in the presence of bicuculline and TTX. Application of 100 μM ATP suppressed the L-IPSC reversibly. The L-IPSC was completely eliminated by 1 μM strychnine (Stry). (**B**) L-IPSCs from an OFF-GC, showing that, in the presence of PPADS (PP) (100 μM) and suramin (Sur) (50 μM), application of 100 μM ATP no longer suppressed the current. (**C**) ATP did not change the L-IPSC of an OFF-GC during internal infusion of 3 mM GDP-β-S.

To further explore physiological implication of the ATP-induced suppression of glycine currents in OFF-GCs, we examined the effects of ATP on glycine receptor-mediated L-IPSCs in retinal slice preparations. Whole cell light responses were recorded from 48 OFF-GCs. In 22 of these cells light-induced responses were completely blocked by co-application of bicuculline and TTX (see Methods for details) and no glycine receptor-mediated L-IPSCs could be recorded. In the remaining 26 GCs, glycine receptor-mediated L-IPSCs were recorded, which were abolished by co-application of 1 μM strychnine. As shown in Fig. 8A, perfusion of 100 μM ATP significantly suppressed the glycine receptor-mediated L-IPSC and the response returned to the control level after washout. Similar results obtained in 8 out 9 OFF-GCs tested, and the average peak amplitudes of the L-IPSCs following ATP application were reduced to 61.8 ± 2.19% of control (*P* < 0.001). For the remaining one cell, ATP had no effect on the currents.

We also tested whether P2Y receptors mediated the ATP-induced suppression of glycine receptor-mediated L-IPSCs in OFF-GCs. Bath application of PPADS (100 μM) and suramin (50 μM) hardly changed L-IPSCs (90.4 ± 11.1%, n = 6, *P* > 0.05), as illustrated by a representative example from an OFF-GC (Fig. 8B). In the presence of PPADS and suramin, the current peak amplitudes obtained after an 8-min perfusion of ATP were 97.1 ± 9.97% of those obtained before ATP perfusion. Moreover, during internal infusion of 3 mM GDP-β-S, ATP did not change the L-IPSCs (100.0 ± 4.23%, n = 5, *P* > 0.05; Fig. 8C).

## Discussion

ATP has been found to regulate both voltage- and ligand-gated channels in central neurons^4^,^5^,^6^,^7^,^8^,^9^,^10^. As far as ligand-gated channels are concerned, NMDA receptors are the only ones which are reported to be modulated by ATP. In the layer V pyramidal neurons of rat prefrontal cortex, for instance, ATP enhances NMDA responses via P2Y_2_ activation^6^.

In the retina it has been previously reported that ATP released from Müller cells and retinal neurons modulates the activity of GCs. Newman (2003, 2004) shows that ATP released from rat Müller cells could mediate interaction between these cells and GCs, suggesting that Müller cells contribute to information processing in the inner retina. Such neuromodulatory actions of ATP on GCs, however, are not due to the activation of P2 receptors, but may be resulted when neuronal adenosine receptors (P1 receptors) are activated by adenosine, which is hydrolyzed from ATP. In the mouse retina Kaneda *et al*.^41^ show that ATP differentially modulates ON-GCs and OFF-GCs, but these authors did not identify the P2 receptor subtypes mediating this modulation of ATP. This work demonstrated, for the first time, that ATP suppresses glycine currents via P2Y receptors. This effect of ATP is neither mediated by P2X receptors (Fig. 3B) nor by P1 receptors (Fig. 2D,E), which is quite different from the effect of ATP released from Müller cells on a subset of GCs that is mediated by P1 receptors. Among the P2Y subtypes, it seems likely that P2Y_1,11_ may be involved, as evidenced by the fact that MRS2365/NF546 induced suppression of glycine currents (Fig. 3E,F). The involvement of P2Y_1,11_ was further suggested by the partial blockade of the ATP effect by the antagonists (MRS2500 and NF157) of these receptor subtypes (Fig. 3C,D). Furthermore, the partial blockade due to either MRS2500 (2 μM) or NF157 (50 μM) raises a possibility that the subtypes (P2Y_2,4,6_) other than P2Y_1,11_ could be also involved. In fact, P2Y_2,4,6_, just like P2Y_1,11_, are also mainly coupled to G_q/11_^57^. The involvement of the P2Y_6_ subtype seems unlikely since it only responds to UDP and UTP, but not ATP^44^,^45^. Whether P2Y_2,4_ may work together with P2Y_1,11_ to mediate the ATP effect remains to be further explored when antagonists for these subtypes are available. Modulation by ATP of glycine responses of retinal GCs observed in this work should be the first report about purinoceptor-mediated modulation of strychnine-sensitive glycine receptors, not only in the retina, but also in the CNS. In the inner retina cholinergic ACs may most likely be the cell type that releases ATP acting on OFF-GCs^26^,^27^.

By pharmacological dissections, we provided evidence showing that a distinct PI-PLC/PKC signaling pathway, following P2Y receptor activation, may be responsible for the ATP effect on glycine responses of OFF-GCs. Actually, this signaling pathway in P2Y_1,2,4,6,11_ receptor-mediated effects has been demonstrated in both neurons and non-neuronal cells^46^,^52^,^55^,^56^,^63^. Furthermore, the ATP effect on glycine currents is dependent on calcium released from intracellular stores via the IP_3_-sensitive pathway. This is consistent with the observation that activation of P2Y receptors by ATP induces IP_3_-mediated calcium release in astrocytes and spinal dorsal horn^44^,^64^,^65^. Consistently, Ca^2+^-dependent PKC (possibly PKCα and β1 isozymes) was involved in the ATP effect (Fig. 7). This is the first work reporting the intracellular signaling pathway, which is schematically depicted in Fig. S1, responsible for the modulation of ligand-gated channels caused by the activation of P2Y receptors.

It is of interest that glycine responses of rat GCs could be modulated due to activation of other G-protein-coupled receptors via a totally different signaling pathway. In isolated rat retinal GCs melatonin activates the G_i/o_ protein-coupled MT2 receptor, thus potentiating glycine responses of rat GCs through a PC-PLC/Ca^2+^-independent PKC signaling pathway^66^.

Glycine is predominantly released by narrow-field ACs, including AII ACs, and these glycinergic ACs mediate the crossover inhibition between ON and OFF pathways^30^,^31^,^32^,^33^,^34^,^35^,^36^,^37^,^38^,^39^, initiated in the ON pathway, that provides an inhibitory conductance to OFF-GCs by controlling glutamate release from presynaptic OFF cone bipolar cells and directly shapes temporal properties of light-evoked responses of OFF-GCs^32^,^35^,^36^,^40^. The suppression by ATP of glycine responses of OFF-GCs suggests that ATP weakens the crossover inhibition, thus resulting in a regulation of spike patterns in OFF-GCs.

There is evidence, showing that ligand-gated receptors may interact with each other, which is mediated either through direct protein-protein interaction^8^,^67^ or via protein phosphorylation^60^,^66^. This direct interaction occurs between P2X and GABA_A_ receptors in neurons in the ventromedial nucleus of the hypothalamus^67^ and between P2Y and NMDA receptors in layer V pyramidal neurons of the prefrontal and parietal cortex^8^. The ATP effect on glycine responses of OFF-GCs should not be a consequence of such a direct crosstalk between P2Y and glycine receptors, as the effect was abolished by G-protein inhibitors (Fig. 4). Nevertheless, the possibility that a direct crosstalk between P2X and GABA_A_ receptors in OFF-GCs could not be ruled out. If this were the case, it would provide a sophisticated way in that the ATP-induced modulation of inhibitory inputs from ACs, mediated respectively by glycine and GABA receptors, comes into play in two different manners, by activating two distinct purinoceptor subtypes: P2Y and P2X.

## Methods

### Ethical approval

All animal protocols were performed in accordance with the National Institutes of Health Guide for the Care and Use of Laboratory Animals and were approved by Animal Care and Use Committee of Shanghai Medical College, Fudan University. Male albino rats (Sprague-Dawley, 15–18 days of age) were used in this study. During this study, all efforts were made to minimize the number of animals used and their pain and discomfort.

### Retinal slice preparations

Retinal slices were prepared following the procedures described previously^68^, with minor modifications. Briefly, following deep anesthesia with 25 mg/ml urethane, the eyes were enucleated, and the retinas were removed. The isolated retinas were vertically cut into 200 μm-thick slices in Ringer’s using a manual cutter (ST-20, Narishige, Tokyo, Japan). The slices were transferred into a recording chamber with the cut side up and held mechanically in place by a grid of parallel nylon strings glued onto a U-shape frame of platinum wire. They were then viewed through a fixed-stage upright microscope (BX51WI, Olympus, Tokyo, Japan) equipped with a 60X water-immersion ceramic objective and DIC optics. Unless described otherwise, retinal slices were perfused continuously with oxygenated and carbogen-bubbled Ringer’s, which contained (in mM) NaCl 125, KCl 2.5, CaCl_2_ 2, MgCl_2_ 1, NaH_2_PO_4_ 1.25, NaHCO_3_ 25, and glucose 15. While recording glycine-induced currents, the extracellular solution was supplemented with CNQX (10 μM), D-AP5 (50 μM), bicuculline (10 μM) and TTX (0.5 μM) to block AMPA-, NMDA-, GABA_A_-receptor-mediated components and voltage-gated sodium channels, respectively. These preparations were used for all the experiments except for calcium imaging.

### Preparation of isolated retinal GCs

The GCs were acutely dissociated from rat retinas by enzymatic and mechanical methods as previously described^69^ with minor modifications. In brief, animals were deeply anaesthetized and the retinas were removed quickly and incubated in oxygenated Hanks’ solution containing (in mM): NaCl 137, NaHCO_3_ 0.5, NaH_2_PO_4_ 1, KCl 3, CaCl_2_ 2, MgSO_4_ 1, HEPES 20, sodium pyruvate 1 and glucose 16, adjusted to pH 7.4 with NaOH. The retinas were then digested in 5–7 mg/ml papain (Worthington Biochemical, Freehold, NJ, USA) containing Hanks’ solution, supplemented with L-cysteine and bovine serum albumin (0.2 mg/ml for each) for 30 min at 33 °C. The solution was bubbled continuously with 100% O_2_. After several rinses in Hanks’ solution, the retinas were mechanically dissociated by gently triturating with fire-polished Pasteur pipettes, and cell suspension was plated onto a culture dish mounted on an inverted microscope (IX 70, Olympus). Isolated GCs were only used for calcium imaging experiments.

### Whole-cell patch-clamp recording

Whole-cell membrane currents of GCs, clamped at −60 mV, were recorded with pipettes of 6–8 MΩ resistance in voltage-clamp modes filled with the internal solution containing (in mM) CsCl 120, CaCl_2_ 1, MgCl_2_ 2, EGTA 10, HEPES 10, ATP-Mg 2, GTP-Na 0.4, NaCl 5 and phosphocreatine 10; adjusted to pH 7.2 with CsOH. Pipettes were mounted on a motor-driven micromanipulator (MP-285, Sutter, Novato, CA, USA), and connected to an EPC10 patch clamp amplifier (HEKA, Lambrecht, Germany). Fast capacitance was fully cancelled and cell capacitance was partially cancelled by the circuits of the amplifier as much as possible. Sixty percent of the series resistance was compensated. Data were acquired at a sampling rate of 5 kHz, and then stored for further analysis. Drug-containing extracellular Ringer’s was either locally applied through a puff pipette (tip diameter ~2 μm), using a pressure micro-injector (PMI-100, DAGAN, Minneapolis, MN, USA), which applied a pressure of 35 kPa (5 p.s.i.) to the top of the pipette, or administrated in bath medium through another inlet by gravity, depending on the purpose of an experiment. Some drugs (GDP-β-S, GPAnt-2a, U73122, D609, mastoparan, BAPTA, heparin, xestospongin-C, ryanodine, Bis IV, Gö6976, cAMP, cGMP, Rp-cAMP and KT5823) were dialyzed into neurons after membrane rapture by including them into the patch electrodes. All experiments were performed at room temperature (20–25 °C).

Electrophysiological recordings of light-evoked responses were performed on retinal slices. Dark-adapted (3 h) rats were deeply anaesthetized and retinal slices were prepared under dim red illumination. Slices were transferred to a recording chamber and superfused constantly with oxygenated bicarbonate-buffered Ringer’s at 30–32 °C. The pipette solution consisted of (in mM): CsCH_3_SO_3_ 120, TEA-Cl 10, Hepes 10, CaCl_2_ 0.1, EGTA 1, phosphocreatine 12, ATP-Mg 3, GTP-Na 0.5; adjusted to pH 7.2 with CsOH. Whole cell light-evoked glycine receptor-mediated IPSCs of GCs were recorded with an EPC 10 amplifier. The cell was held at 0 mV. Bicuculline (10 μM) and TTX (0.5 μM) were added to the perfusion solution to block GABA_A_ receptor and voltage-gated sodium channels, respectively. Light stimuli were generated using an LED (λ = 525 nm). Full-field illumination was delivered from the LED, which was controlled by Pulse software (HEKA Elektronik) and delivered to the retina through the microscope condenser. Photon fluxes on the surface of the superfusion chamber were measured with a linear/log optometer (S350, UDT Instruments, San Diego, CA, USA). Light stimuli of 0.5 μW/cm^2^ were provided for 3 s at 60 s intervals.

GCs were distinguished from displaced ACs in the ganglion cell layer (GCL) according to soma diameters and physiological criteria^68^,^70^,^71^,^72^. ON type GCs (ON-GCs) and OFF-GCs were further identified according to well-established morphological and physiological criteria^30^,^68^,^70^,^73^. Morphologically, ON- and OFF-GCs, revealed by Lucifer yellow, were characterized by their dendrites terminating in proximal and distal parts of the inner plexiform layer, respectively^30^,^68^,^70^. Physiologically, 500-ms negative current injection in the current-clamp mode led to rebound burst firing in the OFF-GCs, but not in the ON-GCs^68^,^70^,^73^. Figure S2 shows an OFF-GC intracellularly stained by Lucifer yellow (A) and its response to a 500-ms negative current injection (B).

### Calcium imaging

Changes in intracellular calcium concentration ([Ca^2+^]_i_) were assessed using the membrane permeable indicator fura-2 AM (Dojindo, Kumamoto, Japan). Fura-2 AM was dissolved in 20% Pluronic F-127 (w/v, DMSO) and added to a chamber that contained Ringer’s, with a final fura-2 AM concentration of 2 μM. Isolated GCs were incubated in the dye solution for 30 min at room temperature and then perfused with dye-free Ringer’s for at least 15 min. Digital fluorescence images were acquired with an inverted microscope (IX-70, Olympus) furnished with a digital CCD camera (ORCA-ER; Hamamatsu Photonics, Shizuoka, Japan). A high-speed continuously scanning monochromatic light source (Polychrome V; Till Photonics, Gräfeling, Germany) was used for the excitations at wavelengths of 340 nm and 380 nm. Fluorescence intensities at both wavelengths (F_340_ and F_380_) were measured every 3–10 s, and images were obtained using PC-based software (C-imaging systems; Hamamatsu Photonic). The ratio between the two images was proportional to [Ca^2+^]_i_ of the cell under study. Prior to an experiment, a background level of fluorescence (attributable to autofluorescence and camera noise) was measured and subtracted from all the obtained data.

### Chemicals

D-AP5, PPADS, suramin, Evans blue, NF157, NF546, MRS2500, MRS2365, ARL67156, GPAnt-2a and ryanodine were purchased from Tocris Bioscience (Ellisville, MO, USA). All other chemicals were obtained from Sigma-Aldrich (St. Louis, MO, USA). Adenosine, U73122, ryanodine, Bis-IV, PMA and Gö6976 were initially dissolved in DMSO for stock and then diluted in solutions to final working concentrations. The final DMSO concentration was less than 0.1%, with no effects on glycine-induced currents of GCs. All other drug solutions were prepared in ion-free water, stored at −20 °C and freshly diluted to the working concentrations using normal solutions.

### Statistical analysis

The data are presented as means ± SEM. Student’s t test (paired data) and one way analysis of variance (ANOVA) followed by *post hoc* Tukey’s tests (multiple comparisons) were used to identify significant differences. In all cases, *P* < 0.05 was considered to be statistically significant.

## Additional Information

**How to cite this article**: Zhang, P.-P. *et al*. Signaling mechanism for modulation by ATP of glycine receptors on rat retinal ganglion cells. *Sci. Rep.* **6**, 28938; doi: 10.1038/srep28938 (2016).

## Supplementary Material

Supplementary Information
