# Supplementary material for: Signaling mechanism for modulation by ATP of glycine receptors on rat retinal ganglion cells
Source: Sci Rep. 2016 Jun 30;6:28938. doi: 10.1038/srep28938 (PMC4928062; doi:10.1038/srep28938)
Supplement: Supplementary Information [file srep28938-s1.pdf]

Signaling mechanism for modulation by ATP of glycine receptors on rat retinal ganglion cells

Ping-Ping Zhang, Gong Zhang, Wei Zhou, Shi-Jun Weng, Xiong-Li Yang and Yong-Mei Zhong

Institutes of Brain Science, State Key Laboratory of Medical Neurobiology and Collaborative Innovation Center for Brain Science, Fudan University, 138 Yixueyuan Road, Shanghai 200032, China.

Fig. S1.

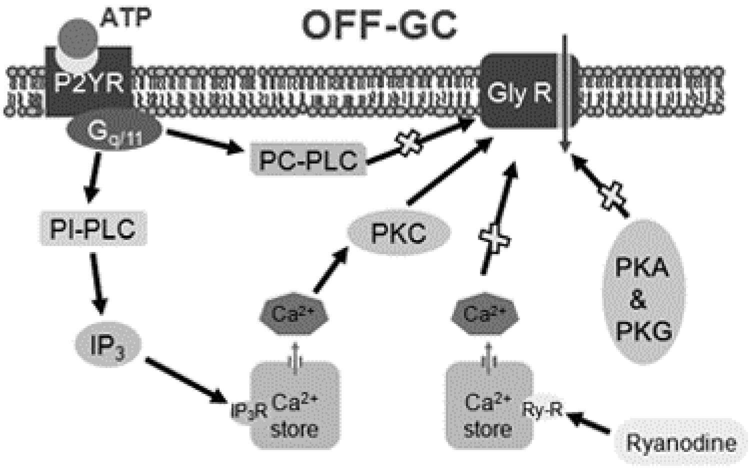

Fig. S1. Schematic diagram showing the signaling pathway that mediates the suppression by ATP of glycine currents in rat retinal OFF-GCs. By activating $G_{q/11}$-coupled $P2Y_1$ and $P2Y_{11}$, ATP suppresses glycine currents via a distinct intracellular PI-PLC/IP_3/Ca^{2+}/PKC signaling pathway. Note that cAMP-PKA and cGMP-PKG signaling pathways are not involved in the effect. GlyR, glycine receptor; IP_3: inositol 1,4,5-trisphosphate; IP_3R: IP_3 receptor; OFF-GC: OFF type ganglion cell; P2YR, P2Y receptor; PC-PLC: phosphatidylcholine-specific phospholipase C; PI-PLC: phosphatidylinositol-specific phospholipase C; PKA: protein kinase A; PKC: protein kinase C; PKG: protein kinase G; Ry-R: ryanodine receptor.

Fig. S2.

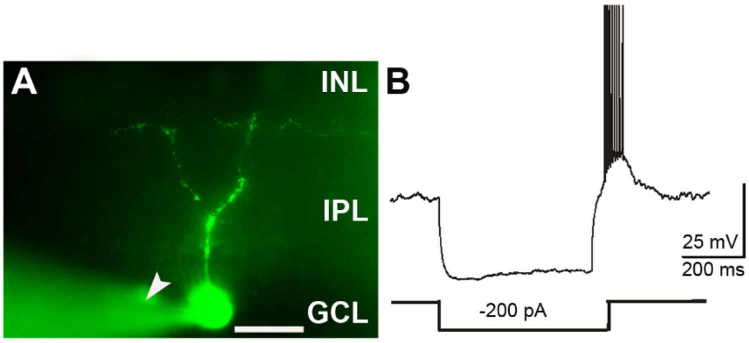

Fig. S2. A rat OFF type ganglion cell (OFF-GC). (A) Fluorescence image of a Lucifer yellow-filled OFF-GC, which possesses dendrite arborization in the distal part of the IPL. The arrowhead points to the recording pipette. INL, inner nuclear layer; IPL, inner plexiform layer; GCL, ganglion cell layer. Scale bar = 20 μm. (B) Voltage response of the cell to a 500 ms negative current step. Note the rebound burst firing at the offset of the current injection.
